# Sirtuin-3 activates the mitochondrial unfolded protein response and reduces cerebral ischemia/reperfusion injury

**DOI:** 10.7150/ijbs.86614

**Published:** 2023-08-21

**Authors:** Xie Xiaowei, Xu Qian, Zhou Dingzhou

**Affiliations:** 1 Department of Neurosurgery, Hunan Provincial People' s Hospital (The First-Affiliated Hospital of Hunan Normal University), Changsha 410005, Hunan Province, People's Republic of China.; 2 Clinical Nursing Teaching and Research Section, The Second Xiangya Hospital, Central South University, Changsha 410011, Hunan Province, People's Republic of China.; 3 Department of Neurology, Haikou City People' s Hospital, Xiangya School of Medicine, Central South University, Haikou 570100, Hainan Province, People's Republic of China.

**Keywords:** Sirt3, UPR^mt^, cerebral I/R injury, Foxo3, Sphk1, mitochondria

## Abstract

Sirtuin-3 (Sirt3) deacetylates several mitochondrial proteins implicated into cerebral ischemia/reperfusion (I/R) injury. The mitochondrial unfolded protein response (UPR^mt^) favors mitochondrial proteostasis during various stressors. Here, we used Sirt3 transgenic mice and a transient middle cerebral artery occlusion model to evaluate the molecular basis of Sirt3 on the UPR^mt^ during brain post-ischemic dysfunction. The present study illustrated that Sirt3 abundance was suppressed in the brain after brain ischemic abnormalities. Overexpression of Sirt3 *in vivo* suppressed the infarction size and attenuated neuroinflammation after brain I/R injury. Sirt3 overexpression restored neural viability by reducing mitochondrial ROS synthesis, maintaining the mitochondrial potential and improving mitochondrial adenosine triphosphate synthesis. Sirt3 overexpression protected neuronal mitochondria against brain post-ischemic malfunction via eliciting the UPR^mt^ by the forkhead box O3 (Foxo3)/sphingosine kinase 1 (Sphk1) pathway. Inhibiting either the UPR^mt^ or the Foxo3/Sphk1 pathway relieved the favorable influence of Sirt3 on neural function and mitochondrial behavior. In contrast, Sphk1 overexpression was sufficient to reduce the infarction size, attenuate neuroinflammation, sustain neuronal viability and prevent mitochondrial abnormalities during brain post-ischemia dysfunction. Thus, the UPR^mt^ protects neural viability and mitochondrial homeostasis, and the Sirt3/Foxo3/Sphk1 pathway is a promosing therapeutic candidate for ischemic stroke.

## Introduction

Cerebral ischemia/reperfusion (I/R) injury results from the restoration of blood flow to the ischemic brain after a stroke, cardiac arrest or traumatic brain injury 1, 2. I/R injury is initiated in the ischemic phase, mainly as a result of deficiency in oxygen or nutrients supply to neural cells 3. However, when blood flow is restored during the reperfusion phase, the ischemic zone of the brain can undergo oxidative stress, inflammatory responses and disordered energy metabolism 4, resulting in blood-brain barrier disruption, neuronal death 5, brain edema and hemorrhaging 6. Considering that cerebral I/R injury has been associated with motor dysfunction, cognitive impairment and even death, it is important to determine the pathological alterations underlying brain I/R.

Recent data reported that mitochondrial abnormality as a key player in brain post-ischemic injury, for several reasons 7, 8. First, interrupted oxidative phosphorylation due to mitochondrial dysfunction can induce neural energy failure 9, 10. Second, mitochondrial damage can cause the release of damage-associated molecular patterns 11, thus triggering an inflammatory response, activating microglia/astrocytes and further exacerbating brain damage. Third, disrupted mitochondrial function increases the generation of ROS, thereby contributing to oxidative injury in the brain 12, 13. Last but not least, poorly structured mitochondria transmit pro-apoptotic signal 14, 15, thus augmenting I/R-induced neuronal death.

Regarding mitochondrial damage, the mitochondrial unfolded protein response (UPR^mt^) is elicited as an endogenous protective system to modify protein folding and prevent abnormal protein accumulation/expression within mitochondria 16, 17. The protective effects of the UPR^mt^ on the brain have been confirmed in a rat traumatic brain injury model 18. Moreover, activation of the UPR^mt^ has been shown to promote mitochondrial proteostasis during adult neurogenesis 19, and to prevent oxidative stress-related neurodegenerative diseases during aging 20. However, the mechanisms of the UPR^mt^ in brain post-ischemic damage are not yet completely investigated 21, 22.

Sirtuin-3 (Sirt3) is a mitochondrial deacetylase that modifies several mitochondrial proteins involved in energy metabolism, ROS production and apoptosis 23, 24. Sirt3 was reported to be involved in the UPR^mt^ in cancer cells 25, and increased Sirt3 expression was associated with the upregulation of UPR^mt^-related genes such as the caseinolytic mitochondrial matrix peptide proteolytic subunit (*CLPP*), 60-kDa heat shock protein (*HSP60*) and Lon peptidase 1 (*LONP1*) in breast cancer 26. In the heart, Sirt3 was found to trigger the mRNA expression of UPR^mt^-related genes by the AMPK pathway, thus improving metabolic remodeling and reducing cardiac hypertrophy 27. Considering that Sirt3 activity is beneficial during brain post-ischemic dysfunction 28, 29, we wondered whether Sirt3 overexpression restrain reperfusion-caused brain abnormalities through eliciting the UPR^mt^.

Sphingosine kinase 1 (Sphk1) is the rate-limiting enzyme in the conversion of sphingosine into sphingosine-1-phosphate, an important regulator of mitochondrial lipid metabolism. Following mitochondrial stress, Sphk1 translocates to the outer mitochondrial membrane, where it increases sphingosine-1-phosphate production and ultimately activates the UPR^mt^ 30, 31. The protein stability of Sphk1 is determined by forkhead box O3 (Foxo3), and the degradation of Foxo3a is prevented by deacetylation 32. Given that Sirt3 is a mitochondrial deacetylase, in this study we investigated whether Sirt3 could induce the UPR^mt^ through the Foxo3a/Sphk1 pathway in order to reduce cerebral I/R injury.

## Materials and Methods

### Animals and models

Sphk1*^Tg^* and Sirt3*^Tg^* mice were established as previously described 33. WT, Sphk1*^Tg^* and Sirt3*^Tg^* mice were treated with 10% chloral hydrate (350 mg/kg, intraperitoneally), and then were subjected to transient MCAO on the right side with a nylon filament. The internal and external carotid arteries were detached, and the middle cerebral artery was blocked with a 4-0 monofilament fiber. The sham surgery was performed similarly, but without MCAO 14. After two hours of ischemia, the fiber was removed for reperfusion, and the mice were monitored for successful model generation based on their observed symptoms. After 24 hours of reperfusion, the mice were euthanized and their brain tissues were collected. The infarction size in the brain was observed using 2% TTC (Sigma) 34.

### Cellular experiments

The N2a cell line was obtained from ATCC (CCL-131^TM^). DMEM containing 10% fetal bovine serum was used to treat N2a cells. To induce N2a cell I/R injury, cells were cultured in an airtight chamber containing 95% N_2_ and 5% CO_2_ for 30 minutes to induce oxygen-glucose deficiency, and then the chamber was sealed with normal oxygen for four hours to induce reoxygenation injury 35. AEBSF (3 μM, cat. no. S7378, Selleck) was used to inhibit the UPR^mt^ in N2a cells. Carbenoxolone (5 mM, cat. no. S4368, Selleck) was used to inhibit the Foxo3 pathway in N2a cells.

### Histology

Fresh brain tissues were firstly treated using 4% paraformaldehyde, followed by embedding using paraffin. Then, 5-μm sections of brain tissues were stained H&E and Nissl staining 36.

### qRT-PCR and western blot analysis

The primers used in the present experiments were showed in supplemental information. Protein extracts were obtained from cells or tissues, quantified with a bicinchoninic acid assay. The separated proteins were moved to polyvinylidene difluoride membranes and then treated with antibodies at 4 ℃ overnight 37. The samples were washed and treated with the corresponding secondary antibodies for one hour 38. After another washing, the membranes were treated with enhanced chemiluminescence reagents for imaging. ImageJ was used to analyze the relative expression of targeted protein bands. The primary antibodies were as follows: Foxo3 (1:1000, ab23683, Abcam), Sphk1 (1:1000, #3297, Cell Signaling Technology) and GAPDH (1:1000, ab8245, Abcam).

### ELISAs

Cells were collected after OGD/R, and ELISAs were performed to analyze the activity of caspase-3 (cat. no. 62218, ThermoFisher), caspase-9 (cat. no. NBP2-75042, Novus), ATP (Mouse Adenosine Triphosphate [ATP] ELISA Kit, cat. no. MOEB2556, AssayGenie), GSH (Mouse Glutathione ELISA Kit, cat. no. MOEB2568, AssayGenie), SOD (Mouse Super Oxidase Dimutase ELISA Kit, cat. no. CSB-E08556m, Cusabio), Complex I (Complex I Enzyme Activity Microplate Assay Kit, cat. no. ab109712, Abcam) and Complex III (cat. no. ab287844, Abcam), according to the manufacturers' instructions and as described previously 39.

### Adenovirus transfection

The Sirt3 and Sphk1 adenoviruses were purchased from Vectro Biolabs 40. AD-293T cells were treated using the adenoviruses (2 plaque-forming units/cell) in serum-free DMEM for one hour. After cell reached 90% confluence, cells were completely lysed (typically after 72 hours). The cells would be destroyed and the maximum number of virions would be freed 41. A multiplicity of infection of 300 in 1 mL of medium was used to infect N2a cells.

### Cell viability determination

Cells at 40% confluency in 24-well plates were transfected with the indicated adenovirus for 48 hours, and then were subjected to OGD/R. MTT assays (Sigma-Aldrich) were performed on a SmartReader^TM^ 96 (Accuris Instruments) at 570 nm to assess cell viability 39. The levels of LDH were measured with a Mouse Lactate Dehydrogenase (LDH) ELISA Kit (cat. no. MODL00786, AssayGenie) 42.

### Immunofluorescence staining

N2a cells were transfected with the adenovirus and then fixed using 4% paraformaldehyde/PBS, followed by incubation with antibodies (Foxo3, 1:1000, ab23683, Abcam; Sphk1, 1:1000, #3297, Cell Signaling Technology) for two hours. Images were observed under an inverted-phase contrast microscope (Olympus) 38.

### Mitochondrial membrane potential and mitochondrial ROS staining

The membrane-permeant JC-1 dye was applied to evaluate the changes in the mitochondrial potential 22. In brief, JC-1 (5 μM, cat. no. T3168, ThermoFisher) was used to incubate with N2a cells and then observed under an inverted-phase contrast microscope (Olympus) 40. To capture the production of mitochondrial ROS, N2a cells were treated with MitoSOX^TM^ (10 μM, cat. no. M36008, ThermoFisher) for 20 minutes and then observed under an inverted-phase contrast microscope (Olympus) 43.

### Statistical analysis

Data are shown as the mean ± SEM and all the experiments were treated at least three times. Student's *t-*tests for comparisons of two group and one-way analysis of variance for comparisons of multiple groups were performed using GraphPad Prism 7 software (GraphPad Software, San Diego, CA, USA). P values < 0.05 were considered statistically significant.

## Results

### Sirt3 overexpression attenuates brain I/R injury

**Figure 1:**
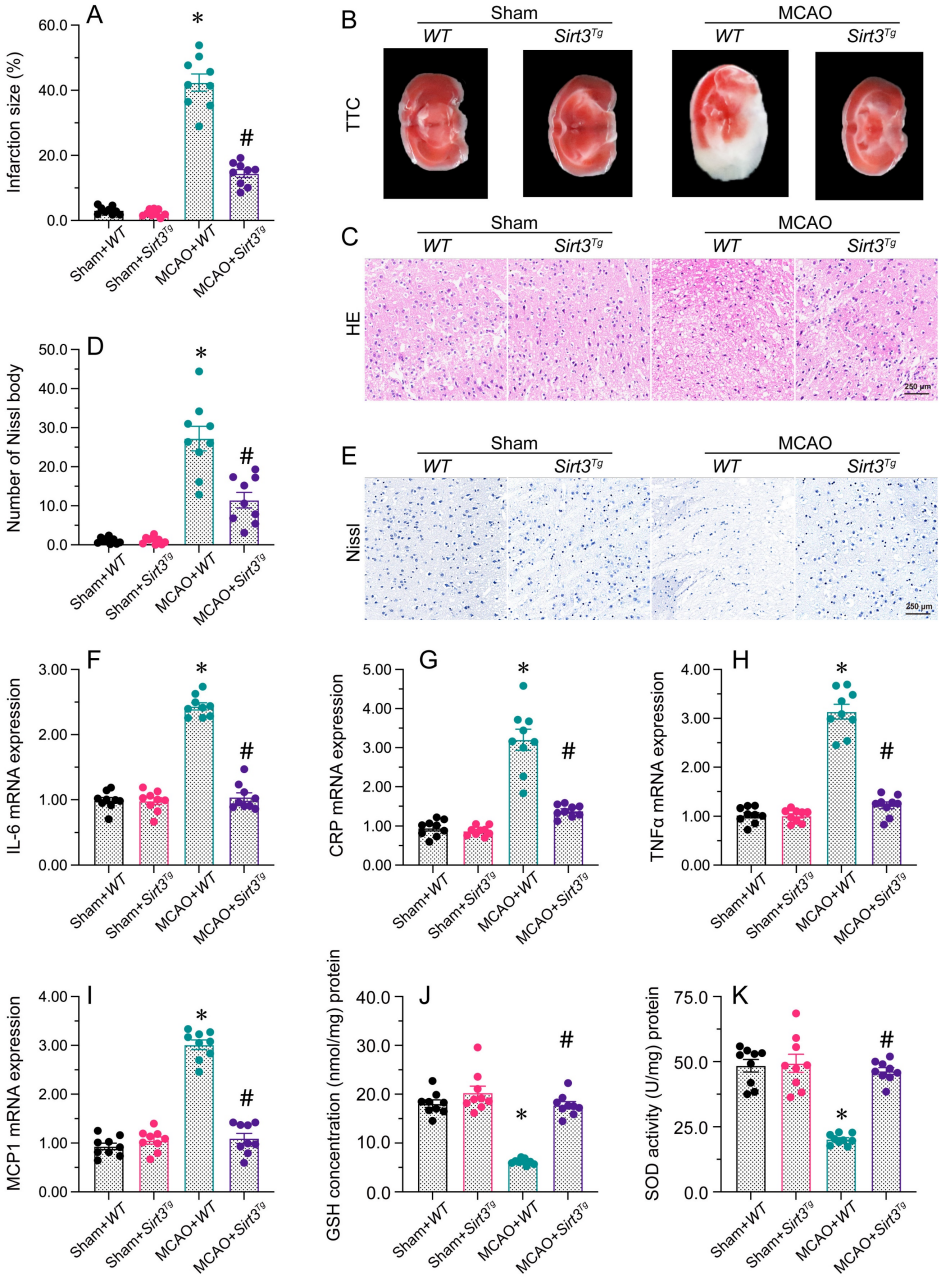
** Sirt3 overexpression attenuates cerebral I/R injury.** WT and Sirt3*^Tg^* mice were subjected to transient MCAO. **A, B.** TTC staining was used to observe the infarction size in the brain after MCAO. **C.** H&E staining was used to detect histological alterations in the brain after MCAO. **D, E.** Nissl staining was used to observe the number of Nissl bodies in brain tissues after MCAO. **F-I.** RNA was isolated from brain tissues, and the levels of *IL-6*, *CRP*, *TNFα* and *MCP1* were analyzed. **J, K.** ELISAs were used to detect GSH and SOD activity levels in brain tissues after MCAO. *p<0.05 vs. sham+WT group, #p<0.05 vs. MCAO+WT group.

To illuminate the molecular basis of Sirt3 on brain I/R injury, Sirt3 expression was assessed by western blotting in the brain in a mouse middle cerebral artery occlusion (MCAO) model. Results exhibited that the transcription of Sirt3 in the brain was markedly suppressed by MCAO (Figure 1A). ELISA further confirmed that Sirt3 activity in the brain was reduced after MCAO treatment.

To determine whether normalizing Sirt3 expression could reduce the vulnerability of the brain to I/R injury, we next subjected Sirt3 transgenic (Sirt3*^Tg^*) mice to MCAO, and compared their infarct sizes with those of wild-type (WT) mice. Staining with TTC revealed that MCAO drastically promoted infarction in the brain tissues of WT mice, but not of Sirt3*^Tg^* mice (Figure 1A). H&E staining of the infarct zone showed that the WT tissues had a porous appearance, and the neuronal nuclei were deeply stained. Moreover, the penumbral tissue was swollen, microglial cells were more prevalent and vascular hyperplasia was detected in WT brain tissues following MCAO. Interestingly, these histological alterations were not detectable in Sirt3*^Tg^* mice subjected to MCAO. In addition, Nissl staining of brain tissues demonstrated that Nissl bodies were rapidly reduced after MCAO in WT mice. The above phenotypic alterations were relieved in Sirt3*^Tg^* mice.

Next, we performed qRT-PCR analyses, which detected that MCAO elevated the mRNA expressions of *IL-6*, *CRP*, *TNFα* and *MCP1* in WT brain tissues. However, overexpression of Sirt3 prevented this pro-inflammatory response. Moreover, the activities of deoxidation enzymes were significantly downregulated after MCAO in WT brain tissues, while Sirt3 overexpression increased the anti-oxidative capacity of the brain following MCAO. Thus, Sirt3 overexpression was sufficient to ameliorate cerebral I/R injury.

### Sirt3 maintains neuronal viability and reduces OGD/R-caused cell death

A drop in cell viability and an increased rate of apoptosis are thought to augment the brain post-ischemic dysfunction. Therefore, next work is to figure out Sirt3 overexpression could increase neural viability. Neuro-2a (N2a) cells were transfected with a Sirt3 overexpression-adenovirus (Ad-Sirt3) or control overexpression-adenovirus (Ad-Ctrl), and then were treated with oxygen-glucose deprivation and reoxygenation (OGD/R). A MTT assay illuminated that cell viability was reduced by OGD/R, whereas Ad-Sirt3 transfection maintained cell viability following OGD/R. Similarly, a LDH release experiment demonstrated that OGD/R increased LDH leakage from Ad-Ctrl cells, whereas Ad-Sirt3 treatment reversed this change. Trypan blue staining indicated that OGD/R elevated the ratio of apoptotic N2a cells in the Ad-Ctrl group; this increase was lessened in the Ad-Sirt3 group. An ELISA revealed that caspase-3 activity was rapidly elevated by OGD/R in Ad-Ctrl cells, but was attenuated to near-physiological condtions in Ad-Sirt3 cells. We also observed that OGD/R enhanced the transcription of fatal genes such as *Bax* and *Bad*, while it markedly reduced the levels of pro-survival genes such as *Bcl-2* and *c-IAP* in Ad-Ctrl cells. Transfection of Ad-Sirt3 suppressed *Bax/Bad* transcription and increased *Bcl-2/c-IAP* levels following OGD/R. Thus, Sirt3 overexpression maintained cell viability and prevented apoptosis in neurons.

### Sirt3 stabilizes mitochondrial function in neurons

Since mitochondrial dysfunction is regarded as a contributor to neural apoptosis after brain I/R injury 44, 45, we next investigated whether Sirt3 was able to favor mitochondrial performance during brain I/R injury. *In vitro*, the mitochondrial potential was rapidly inhibited in N2a cells exposed to OGD/R; however, Sirt3 overexpression sustained the mitochondrial potential despite treatment with OGD/R. OGD/R also induced mitochondrial ROS production, whereas this change was attenuated in Ad-Sirt3-transfected cells. Considering the importance of mitochondria for adenosine triphosphate (ATP) production, we used an ELISA to measure ATP levels. We found that OGD/R interrupted ATP synthesis in Ad-Ctrl-transfected N2a cells, but not in Ad-Sirt3-transfected N2a cells. Cellular ATP production depends upon mitochondrial respiration. OGD/R notably repressed the content of mitochondrial respiratory complexes I and III, while Ad-Sirt3 transfection enhanced them in OGD/R-treated cells.

### Sirt3 overexpression protects neuronal viability and mitochondrial function through the UPR^mt^

**Figure 4:**
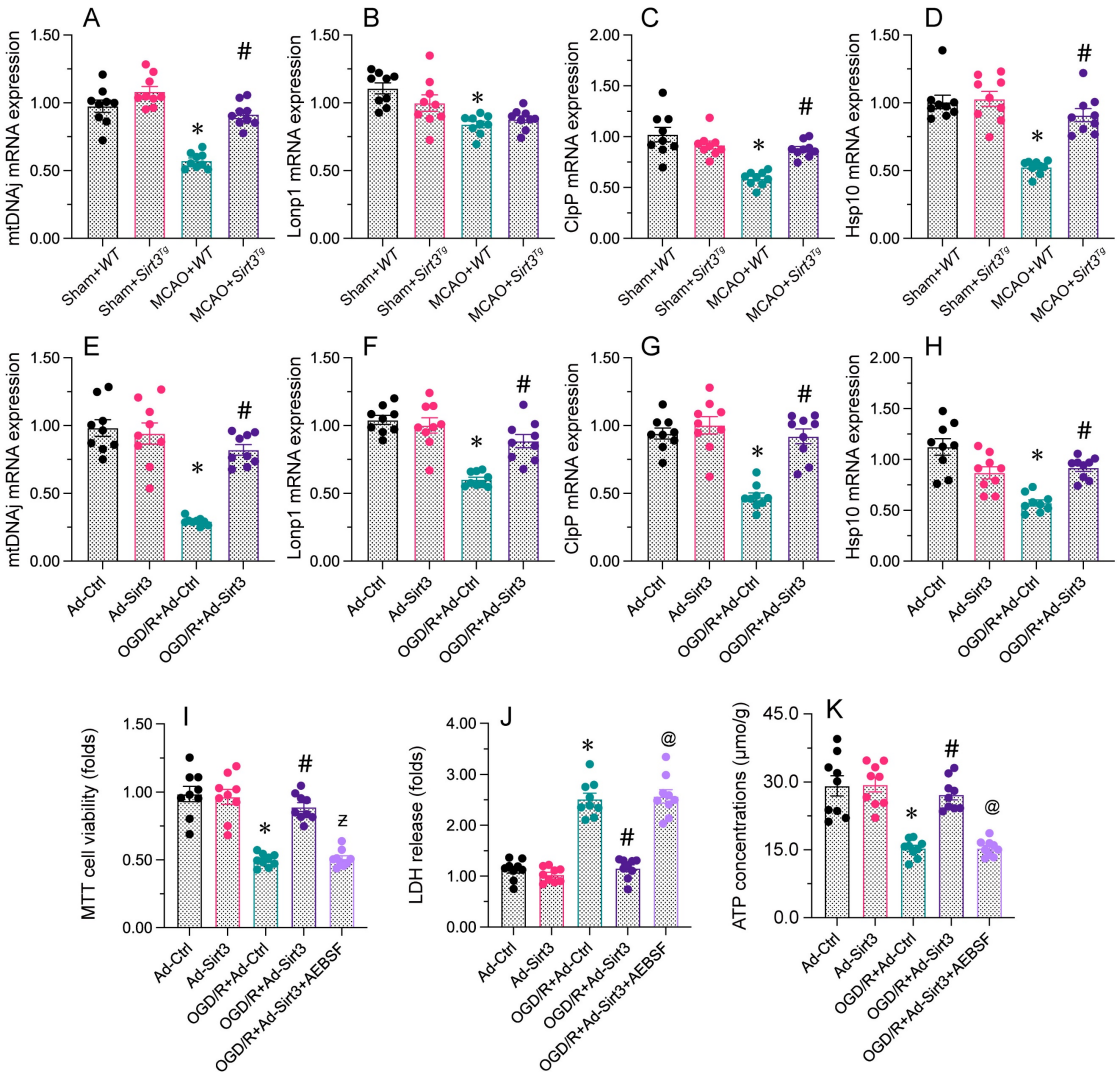
** Sirt3 overexpression protects neuronal viability and mitochondrial function by activating the UPR^mt^.** WT and Sirt3*^Tg^* mice were subjected to transient MCAO. N2a cells were transfected with Ad-Ctrl or Ad-Sirt3, and then were subjected to OGD/R to simulate cerebral I/R injury *in vitro*. **A-D.** RNA was collected from brain tissues, and *mtDNAj*, *Lonp1*, *ClpP* and *Hsp10* levels were measured using qPCR. **E-H.** The mRNA levels of *mtDNAj*, *Lonp1*, *ClpP* and *Hsp10* were evaluated using qRT-PCR in N2a cells subjected to OGD/R. **I.** AEBSF was used to inhibit the UPR^mt^, and cell viability was determined with an MTT assay. **J.** AEBSF was used to inhibit the UPR^mt^, and then an LDH release assay was used to measure LDH levels in media from N2a cells. **K.** AEBSF was used to inhibit the UPR^mt^, and then the concentration of ATP in N2a cells was detected with an ELISA. *p<0.05 vs. Ad-Ctrl group or sham+WT group, #p<0.05 vs. OGD/R+Ad-Ctrl group or MCAO+WT group, @p<0.05 vs. OGD/R+Ad-Sirt3 group.

To assess whether Sirt3 protected neuronal mitochondria by activating the UPR^mt^, we next measured the mRNA expression of UPR^mt^-related genes in brain tissues from WT or Sirt3*^Tg^* mice. Compared with the sham treatment, MCAO treatment reduced the mRNA expression of *mtDNAj*, *ClpP*, *Lonp1* and *Hsp10* in WT mice (Figure 4A); however, these differences were not observed in Sirt3*^Tg^* mice. Similar results were obtained in N2a cells *in vitro*: OGD/R inhibited the mRNA expression of *mtDNAj*, *ClpP*, *Lonp1* and *Hsp10*, whereas Ad-Sirt3 transfection reversed this effect.

To confirm that the UPR^mt^ was responsible for the defensive impacts of Sirt3 on N2a cell viability and mitochondrial capacity, we incubated N2a cells with AEBSF, an inhibitor of the UPR^mt^, before transfecting the cells with Ad-Sirt3. A CCK-8 assay indicated that AEBSF abrogated the protective effects of Ad-Sirt3 on N2a cell viability following OGD/R. An LDH release assay demonstrated that AEBSF elevated LDH release from Ad-Sirt3-transfected OGD/R-treated N2a cells. Regarding mitochondrial function, AEBSF also reduced ATP synthesis following OGD/R in Ad-Sirt3-transfected N2a cells.

### Sirt3 activates the UPR^mt^ in neurons through the Foxo3a/Sphk1 pathway

To understand the mechanism through which Sirt3 activates the UPR^mt^, we focused on the Foxo3a/Sphk1 pathway. Western blotting of brain tissues revealed that Foxo3a and Sphk1 levels were significantly reduced in MCAO-treated WT mice, but were returned to physiological state in MCAO-treated Sirt3*^Tg^* mice. In consonance with the above evidence, immunofluorescence analyses illuminated that OGD/R treatment inhibited Foxo3a and Sphk1 expression in N2a cells, whereas Ad-Sirt3 transfection reversed these effects.

Although the above results demonstrated that Sirt3 activates the Foxo3a/Sphk1 pathway, further investigation was needed to determine whether the Foxo3a/Sphk1 pathway participates in Sirt3-induced UPR^mt^ activation. We used carbenoxolone, a Foxo3 inhibitor, to suppress the Foxo3a/Sphk1 pathway. Carbenoxolone prevented Sirt3 overexpression from upregulating *mtDNAj*, *ClpP*, *Lonp1* and *Hsp10* in N2a cells during OGD/R treatment. These findings suppored that Sirt3 activates the UPR^mt^ in neurons through the Foxo3a/Sphk1 pathway.

### Sphk1 overexpression suppresses brain I/R injury

To assess whether restoration of the Foxo3/Sphk1 pathway would be sufficient to reduce brain I/R injury, we established an MCAO model in Sphk1 transgenic (Sphk1*^Tg^*) mice. The MCAO-induced infarction of brain tissue was attenuated in Sphk1*^Tg^* mice compared with WT mice. Furthermore, H&E histological analysis demonstrated that neuronal nuclei around the infarction were pyknotic and deeply stained after MCAO treatment in WT mice, while these structural abnormalities were alleviated in neurons from Sphk1*^Tg^* mice. Similarly, Nissl staining of brain tissues revealed that MCAO significantly elevated the number of Nissl bodies in WT mice whereas the above changes seem to be imperceptible in Sphk1*^Tg^* mice.

MCAO also upregulated the gene expression of *IL-6*, *CRP*, *TNFα* and *MCP1* in brain tissues from WT mice. However, the above gene transcriptions were ameliorated in Sphk1*^Tg^* mice. Additionally, MCAO treatment reduced the concentrations of oxidation-suppressing factors (GSH and GPX) in WT brains, whereas Sphk1 overexpression attenuated this effect. These results demonstrated that restoring Sphk1 expression was sufficient to attenuate brain I/R injury.

### Impediment of the UPR^mt^ reduces Sphk1 overexpression-caused protection on neurons and mitochondria

Next, we evaluated whether Sphk1 overexpression reduces brain I/R dysfunction by normalizing the UPR^mt^. The present data exhibited that adenoviral overexpression of Sphk1 (Ad-Sphk1) impede OGD/R-mediated cell viability decrease. However, treatment with the UPR^mt^ inhibitor AEBSF abolished the pro-survival effects of Ad-Sphk1. Ad-Sphk1 also suppressed LDH release from OGD/R-treated N2a cells, while AEBSF prevented this effect. Regarding mitochondrial function, Ad-Sphk1 sustained the mitochondrial membrane potential in N2a cells under OGD/R stress, while AEBSF administration abrogated the protective effects of Ad-Sphk1. Moreover, Ad-Sphk1 transfection repressed mitochondrial ROS production in OGD/R-challenged N2a cells, but AEBSF nullified the anti-oxidative capacities of Ad-Sphk1. Lastly, ELISAs showed that caspase-3 and caspase-9 activity levels were augmented upon OGD/R treatment and reduced to near-normal levels following Ad-Sphk1 transfection; however, in N2a cells treated with AEBSF, Ad-Sphk1 failed to inhibit caspase-3/9 activation. Thus, inhibiting the UPR^mt^ neutralized the beneficial impacts of Sphk1 overexpression on neural function and mitochondrial integrity.

## Discussion

Our work highlighted that Sirt3 can attenuate brain I/R injury via normalizing the UPR^mt^ ; and this function is highly relied on the Foxo3/Sphk1 cascade. This observation had several novel findings. First, Sirt3 was depressed by brain post-ischemic injury, and reduced Sirt3 expression was associated with an augmented infarction size in the brain, suggesting that Sirt3 is an endogenous protector against ischemic stroke. Second, overexpression of Sirt3 restored neural viability by normalizing mitochondrial homeostasis, indicating that Sirt3 prevents ischemic stroke by protecting mitochondria. Third, inhibiting the UPR^mt^ abolished the beneficial impacts of Sirt3 overexpression on neural function and mitochondrial performance during brain I/R injury, suggesting that inducing the UPR^mt^ could be an unconventional medicinal way to diminish the vulnerability of mitochondria during brain I/R injury. Fourth, Sirt3 promoted the UPR^mt^ by upregulating the Foxo3/Sphk1 pathway, and enhancing Sphk1 expression was sufficient to protect neurons and their mitochondria against brain I/R injury. This investigation demonstrates that the UPR^mt^ is a critical protective mechanism to maintain neural viability and mitochondrial homeostasis, and suggest that the Sirt3/Foxo3/Sphk1 pathway is a valuable medicinal objective for patients with ischemic brain damage in clinical practice.

The involvement of mitochondrial dysfunction in brain ischemic dysfunction is broadly reported 17, 43, 46-48. MCAO was found to upregulate mitochondrial fission-related proteins, increase mitochondrial fission, augment mitochondrial ROS production and activate mitochondrial apoptosis in the brain 49. Similarly, transmission electron microscopy revealed that MCAO induced mitochondrial fragmentation in brain tissues 50. Disrupted mitochondrial homeostasis was associated with increased NLR family pyrin domain-containing 3 inflammasome levels in the brain through an undefined mechanism during brain ischemic dysfunction 51. Brain post-ischemic damage was also found to induce optic atrophy 1 cleavage at the S1 site, thus contributing to mitochondrial cristae remodeling and mitochondrial membrane rupture in neurons 52.

Considering that mitochondrial dysfunction accelerates cerebral I/R injury, several pharmacological interventions have been introduced to attenuate these pathological effects. Ligustilide was found to mitigate brain ischemic dysfunction by activating Parkin-induced mitophagy 53. Danhong injection, a classical Chinese medicinal approach, was proved to alleviate brain post-ischemic abnormalities by improving mitochondria-dependent neural metabolism 9. Garciesculenxanthone B was explained to reduce limit brain post-ischemic malfunction by enhancing PTEN-induced kinase 1/Parkin-dependent mitophagy 54.

In addition to these pharmacological approaches, several careful studies have examined non-pharmacological therapies to restore mitochondrial homeostasis during cerebral I/R injury. Mitochondrial transplantation surgery was used to deliver new, well-organized mitochondria to the infarcted brain 55. This technique was reported to alleviate mitochondrial DNA damage and increase the mitochondrial membrane potential, thus ameliorating neurobehavioral deficits and reducing the infarct size after cerebral I/R injury 55. Consistent with these findings, delivery of placental mitochondria was revealed to diminish transient brain ischemic anomaly via boosting mitochondrial oxidation-suppressing enzymes levels 56.

The UPR^mt^ maintains mitochondrial proteostasis in response to various cellular stressors 16, 17, 57. The UPR^mt^ is stirred if the mitochondrial protein folding machinery is overwhelmed or impaired, leading to the accumulation of misfolded or unfolded proteins 58. Upon activation, the UPR^mt^ augments the abundance of various molecular chaperones, proteases and other proteins that help to restore proteostasis and promote mitochondrial function 59, 60. The UPR^mt^ is regulated by several transcription factors, including stress-activated transcription factor 1 and hypoxia inducible factor 1α 61, 62, which translocate from the mitochondria to the nucleus where UPR^mt^ genes expression are rapidly elicited.

The beneficial effects of the UPR^mt^ on cerebral diseases have been widely described. Activation of the UPR^mt^ was reported to restore the concentrations of mitochondrial complex I and thus prevent the progression of Alzheimer's disease 63. In subarachnoid hemorrhage, overexpression of GrpE-like 1 was found to enhance mtHSP70 expression, thus activating the UPR^mt^ and accelerating the degradation of abnormal proteins 64. During traumatic brain injury, activation of the UPR^mt^ was shown to maintain the mitochondrial membrane potential, reduce the neuroinflammatory response and inhibit mitochondrial apoptosis 18. Injection of honokiol seem to relieve cognitive impairment in APP/PS2 mice by eliciting the UPR^mt^ 65.

In this work, our data showed that Sirt3 restored UPR^mt^ activity through the Foxo3/Sphk1 pathway. The beneficial impact of Sirt3 on the brain has been described in several previous studies. Upregulation of Sirt3 through melatonin administration was found to ameliorate brain I/R injury via reducing mitochondrial oxidative stress 29. Similarly, treatment with genipin elevated Sirt3 expression and thus improved uncoupling protein 2-dependent mitochondrial metabolism during cerebral I/R injury 66. Sirt3 overexpression activated Wnt/β-catenin and blocked mitochondrial fission, thereby reducing cerebral I/R injury 67. In another study, Sirt3 overexpression inhibited the TLR4/NF-κB pathway and activated the Nrf2/Keap1 pathway, ultimately normalizing the redox response and reducing neuroinflammation during brain ischemic dysfunction 68. On the basis of these observations of our study, Sirt3 downregulation seems to be a biomarker of brain post-ischemic abnormalities, so Sirt3 overexpression is a bright medicative method to prevent brain post-ischemic anomaly.

In sum, our study illustrated the beneficial effects of Sirt3 overexpression during brain I/R injury. Sirt3 overexpression preserved the UPR^mt^ by inducing the Foxo3/Sphk1 pathway in neurons, thereby improving mitochondrial function and reducing neuronal apoptosis. On the basis of our results, augmentation of Sirt3 expression and activation of the UPR^mt^ are encouraging medicative tool for ischemic stroke.

## Supplementary Material

Supplementary primers.

## Figures and Tables

**Figure 2:**
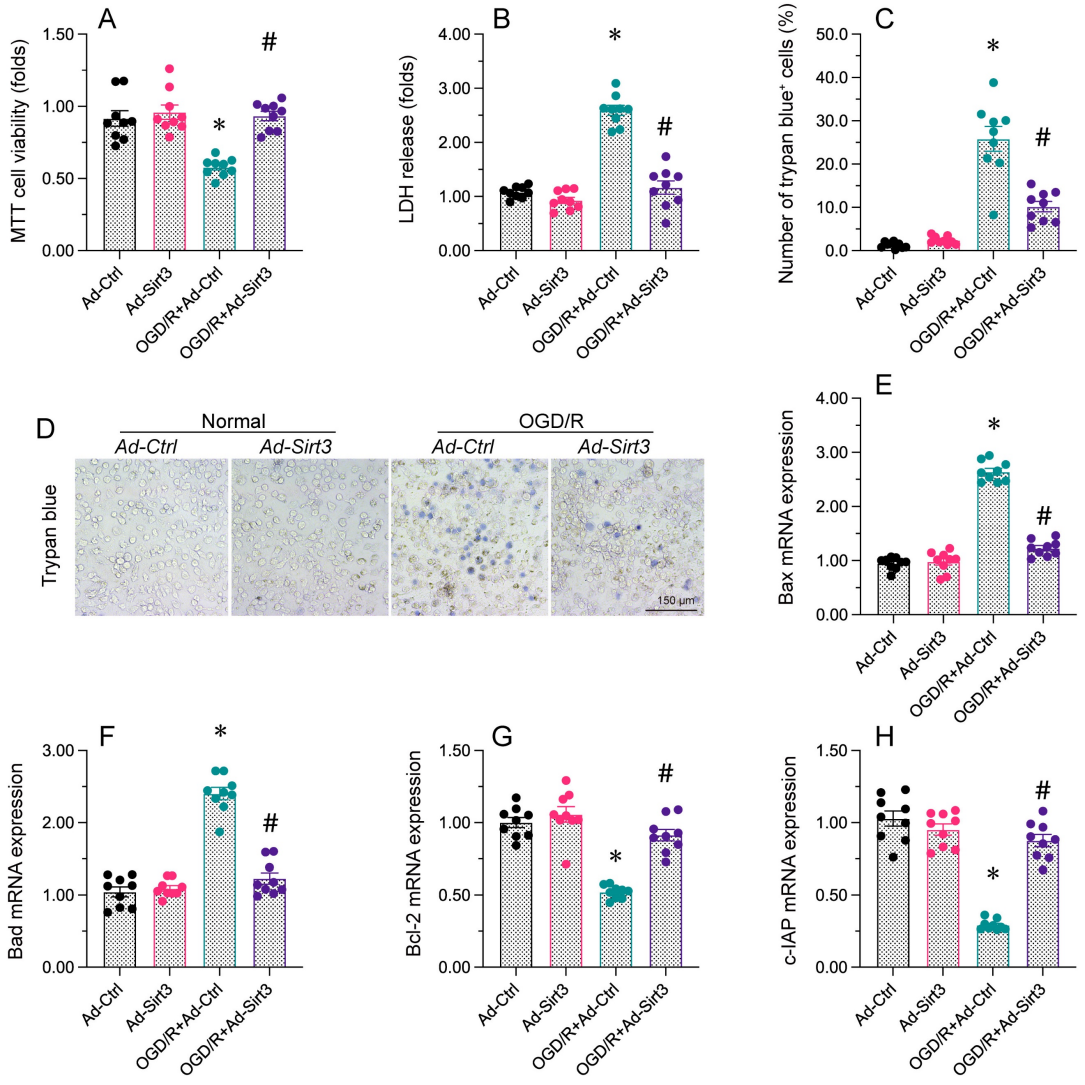
** Sirt3 maintains neuronal viability and reduces OGD/R-induced cell death.** N2a cells were transfected with Ad-Ctrl or Ad-Sirt3, and then were subjected to OGD/R to simulate cerebral I/R injury *in vitro*. **A.** Cell viability was determined with an MTT assay.** B.** An LDH release assay was used to measure LDH levels in media from N2a cells. **C, D.** Trypan blue staining was used to observe the number of apoptotic cells after OGD/R. **E-H.** RNA was isolated from N2a cells, and the levels of *Bax*, *Bad*, *Bcl-2* and *c-IAP* were measured. *p<0.05 vs. Ad-Ctrl group, #p<0.05 vs. OGD/R+Ad-Ctrl group.

**Figure 3:**
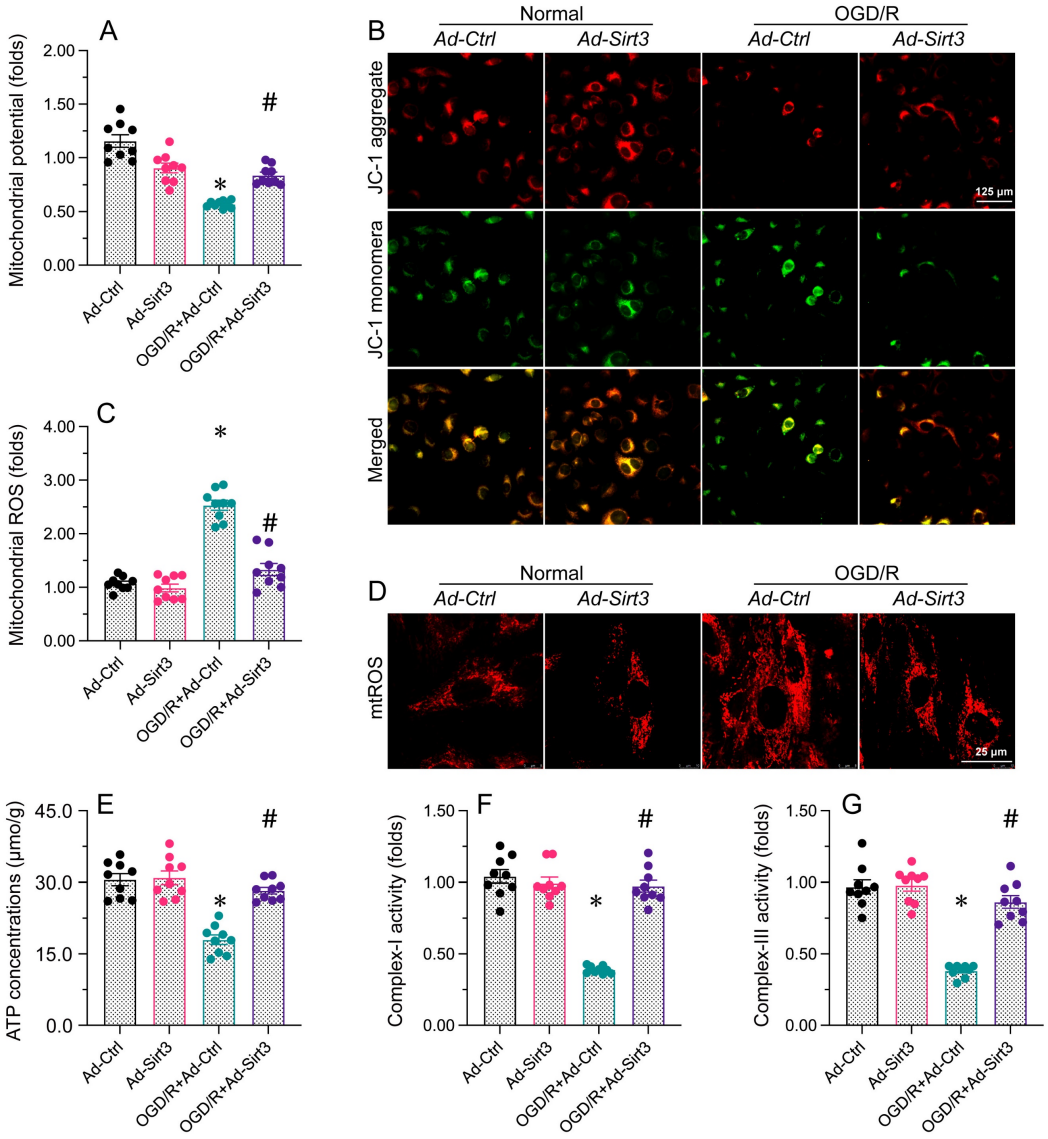
** Sirt3 stabilizes mitochondrial function in neurons.** N2a cells were transfected with Ad-Ctrl or Ad-Sirt3, and then were subjected to OGD/R to simulate cerebral I/R injury *in vitro*. **A, B.** JC-1 probes were used to analyze the mitochondrial membrane potential in N2a cells after OGD/R injury. The red-to-green fluorescence ratio was used to quantify the mitochondrial membrane potential.** C, D.** Immunofluorescence was used to measure the production of mitochondrial ROS. **E.** The concentration of ATP in N2a cells was detected with an ELISA. **F, G.** ELISAs were used to determine complex I and complex III activity levels in N2a cells. *p<0.05 vs. Ad-Ctrl group, #p<0.05 vs. OGD/R+Ad-Ctrl group.

**Figure 5:**
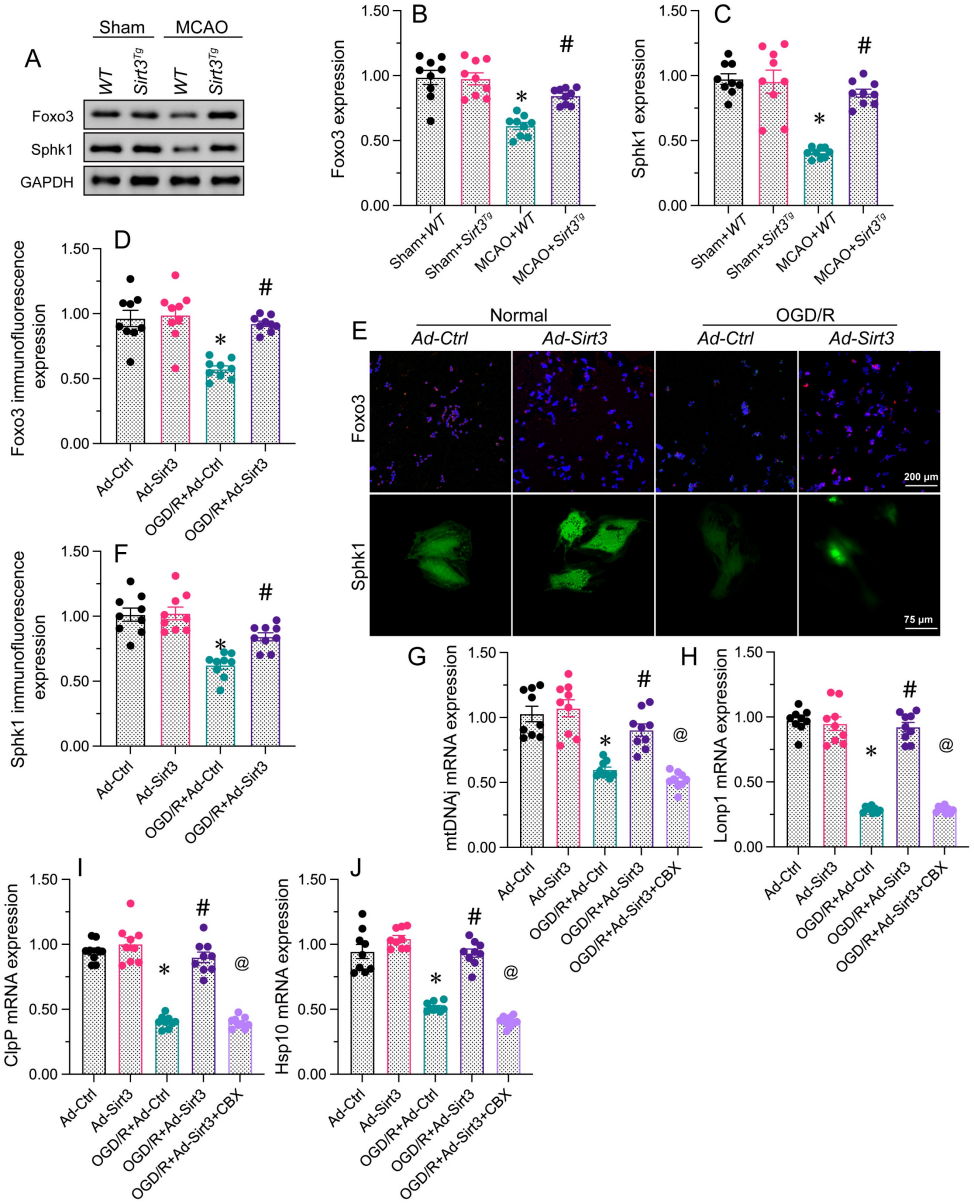
** Sirt3 activates the UPR^mt^ in neurons through the Foxo3a/Sphk1 pathway.** WT and Sirt3*^Tg^* mice were subjected to transient MCAO. N2a cells were transfected with Ad-Ctrl or Ad-Sirt3, and then were subjected to OGD/R to simulate cerebral I/R injury *in vitro*. **A-C.** Proteins were isolated from brain tissues, and Western blotting was used to measure the expression of Foxo3 and Sphk1.** D-F.** Immunofluorescence was used to observe changes in Foxo3 and Sphk1 levels in N2a cells after OGD/R. **G-J.** Carbenoxolone (CBX) was used to inhibit the Foxo3/Sphk1 pathway, and then qRT-PCR was performed to measure *mtDNAj*, *Lonp1*, *ClpP* and *Hsp10* mRNA levels in N2a cells subjected to OGD/R. *p<0.05 vs. Ad-Ctrl group or sham+WT group, #p<0.05 vs. OGD/R+Ad-Ctrl group or MCAO+WT group, @p<0.05 vs. OGD/R+Ad-Sirt3 group.

**Figure 6:**
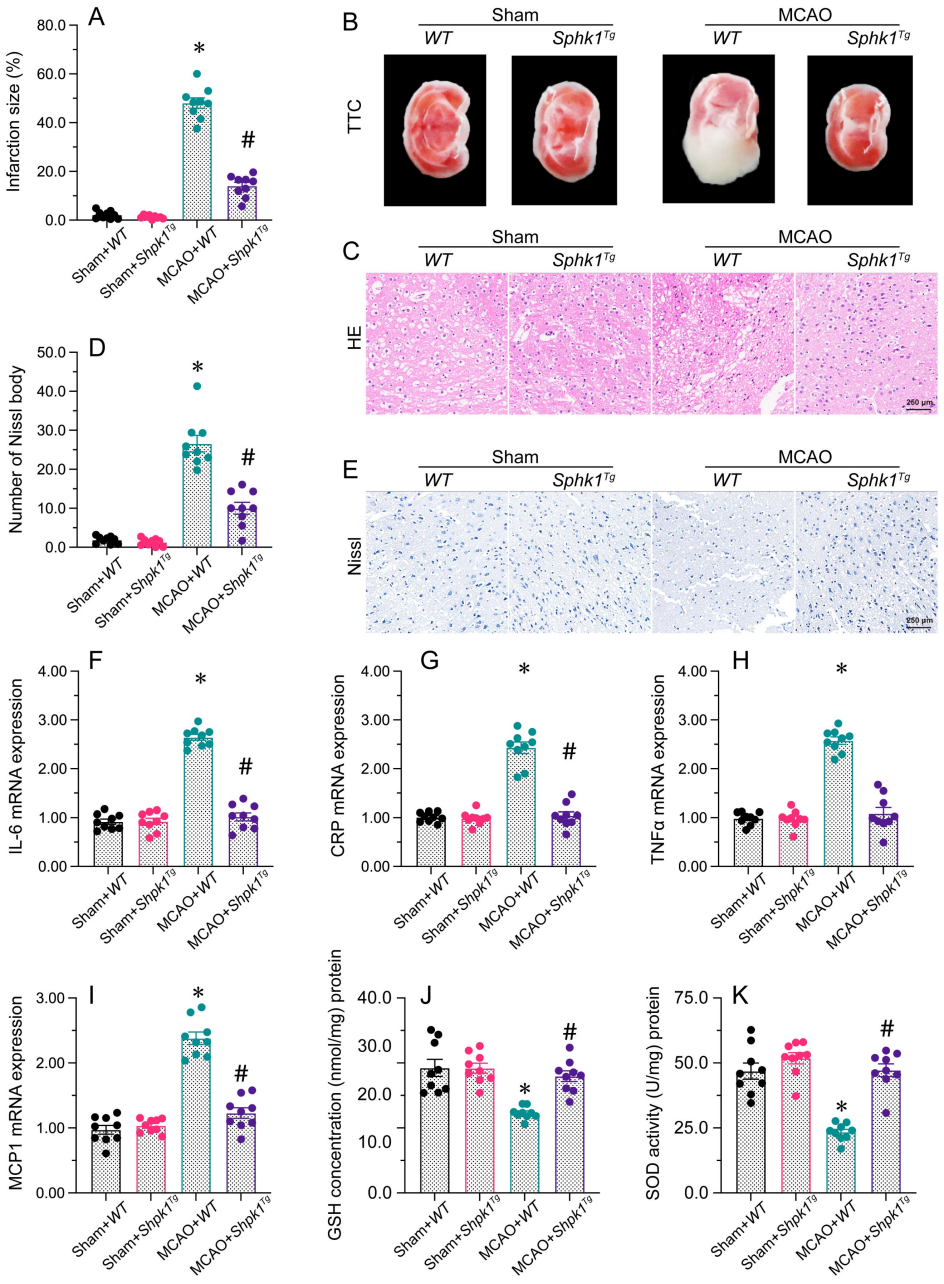
** Sphk1 overexpression protects the brain against I/R injury.** WT and Sphk1*^Tg^* mice were subjected to transient MCAO. **A, B.** TTC staining was used to observe the infarction size in the brain after MCAO. **C.** H&E staining was used to detect histological alterations in the brain after MCAO. **D, E.** Nissl staining was used to observe the number of Nissl bodies in brain tissues after MCAO. **F-I.** RNA was isolated from brain tissues, and the levels of *IL-6*, *CRP*, *TNFα* and *MCP1* were measured. **J, K.** ELISAs were used to detect changes in GSH and SOD activity in brain tissues after MCAO. *p<0.05 vs. sham+WT group, #p<0.05 vs. MCAO+WT group.

**Figure 7:**
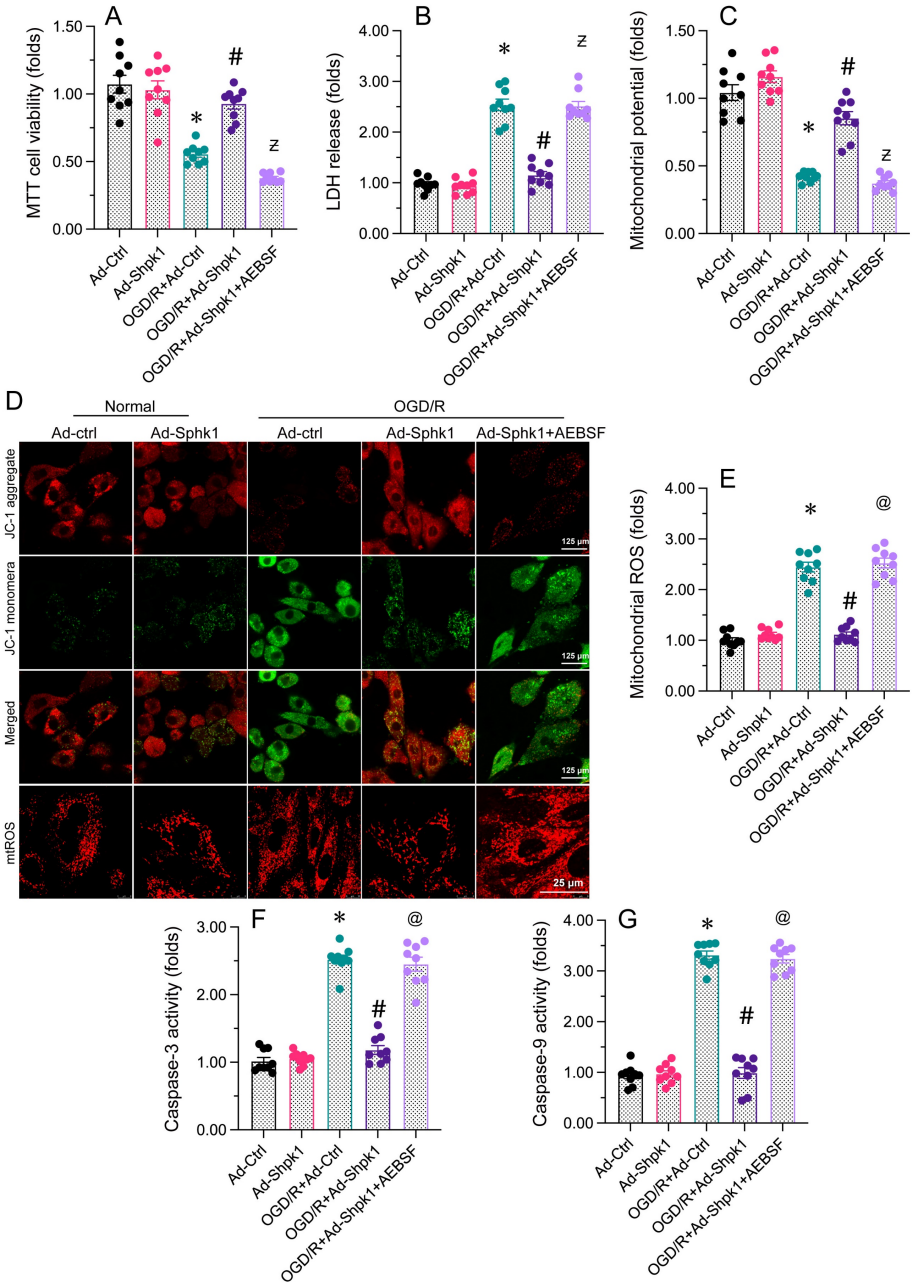
** Inhibiting the UPR^mt^ abolishes the protective effects of Sphk1 overexpression on neurons and their mitochondria.** N2a cells were transfected with Ad-Ctrl or Ad-Sphk1, and then were subjected to OGD/R to simulate cerebral I/R injury *in vitro*. **A.** Cell viability was determined with an MTT assay.** B.** An LDH release assay was used to measure LDH levels in media from N2a cells. **C, D.** JC-1 probes were used to analyze the mitochondrial membrane potential in N2a cells after OGD/R injury. The red-to-green fluorescence ratio was used to quantify the mitochondrial membrane potential.** E.** Immunofluorescence was used to measure the production of mitochondrial ROS. **F, G.** The activity levels of caspase-3 and caspase-9 were determined using ELISAs in N2a cells. *p<0.05 vs. Ad-Ctrl group, #p<0.05 vs. OGD/R+Ad-Ctrl group, @p<0.05 vs. OGD/R+Ad-Sphk1 group.
