# Supplementary material for: Sirtuin-3 activates the mitochondrial unfolded protein response and reduces cerebral ischemia/reperfusion injury
Source: Int J Biol Sci. 2023 Aug 21;19(13):4327–39. doi: 10.7150/ijbs.86614 (PMC10496505; doi:10.7150/ijbs.86614)
Supplement: Supplementary file 1 — Supplementary primers. [file ijbsv19p4327s1.pdf]

Supplemental Information

The primers used in the present experiments were as follows:

mtDNAj, Forward, 5'-AGTCACCCACACAAGCACTG-3', Reverse, 5'-CCAGCCTCTCGCCTATCC-3';

ClpP, Forward, 5'-CACAGACATCGCCATCCA-3', Reverse, 5'-TCCCTCTCCATTGCTGACTC-3';

Lonp1, Forward, 5'-GGTTGAGAATGTAGCCCATGA-3', Reverse, 5'-CGATGATATCCCGAATGGTC-3';

Hsp10, Forward, 5'-GGCCCGAGTTCAGAGTCC-3', Reverse, 5'-TGTCAAAGAGCGGAAGAAACTT-3';

TNFα, Forward, 5'- AGATGGAGCAACCTAAGGTC-3', Reverse, 5'-GCAGACCTCGCTGTTCTAGC-3';

IL-6, Forward, 5'-CAGACTCGCGCCTCTAAGGAGT-3', Reverse, 5'-GATAGCCGATCCGTCGAA-3';

MCP1, Forward, 5'-GGATGGATTGCACAGCCATT-3', Reverse, 5'-GCGCCGACTCAGAGGTGT-3'.
